# Supplementary material for: Oral Supplementation of the Vitamin D Metabolite 25(OH)D3 Against Influenza Virus Infection in Mice
Source: Nutrients. 2020 Jul 5;12(7):2000. doi: 10.3390/nu12072000 (PMC7400405; doi:10.3390/nu12072000)
Supplement: Supplementary file 1 [file nutrients-12-02000-s001.pdf]

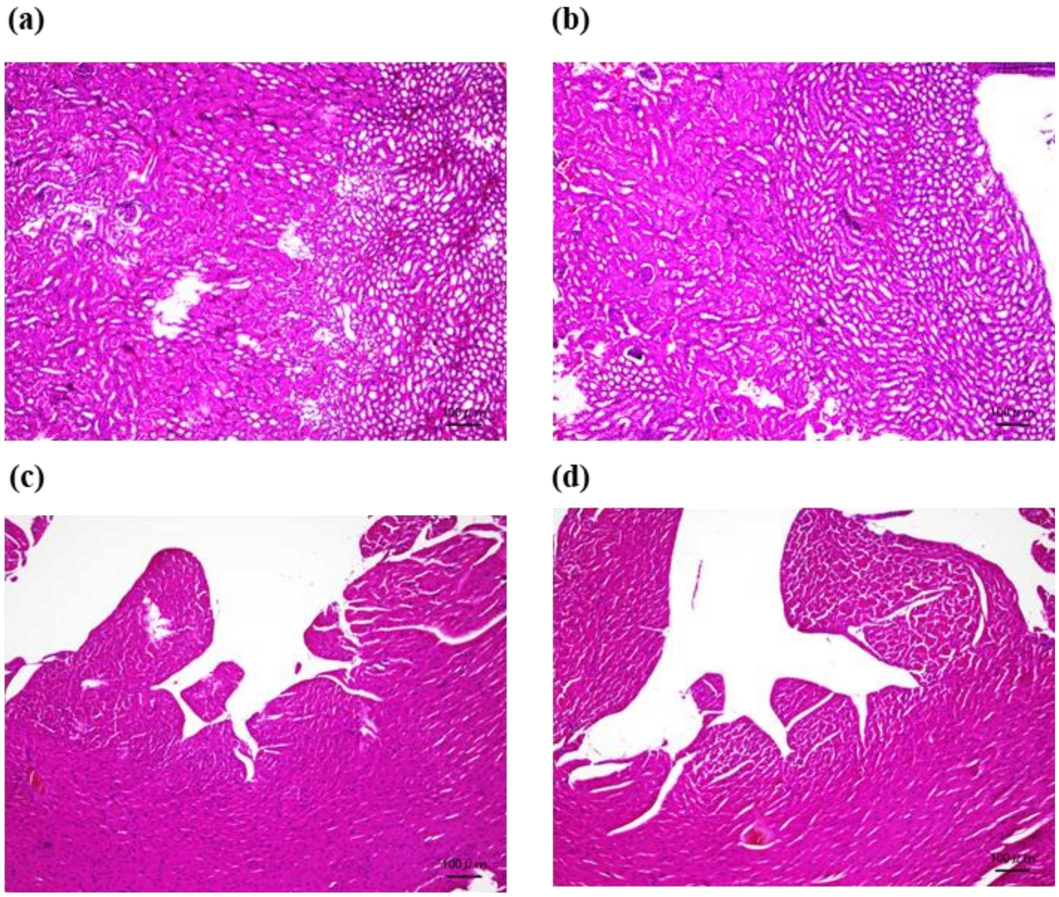

Figure S1. Histopathological findings in mouse kidneys and hearts. Mice were fed for 7 weeks, and kidneys and hearts were sectioned and stained with hematoxylin and eosin. The kidneys of a standard-fed mouse **(a)** and a 25(OH)D_3-fed mouse **(b)**. The hearts of a standard-fed mouse **(c)** and a 25(OH)D_3-fed mouse (**d**).
